# Concerning Inguinal Hernia in the Female Dog

**Published:** 1901-07

**Authors:** Cecil French

**Affiliations:** Washington, D. C.


### THE JOURNAL

OF

### COMPARATIVE MEDICINE AND VETERINARY ARCHIVES.

VOL. XXII. *JULY, 1901.* NO. 7.

### CONCERNING INGUINAL HERNIA IN THE FEMALE DOG.

BY CECIL FRENCH, D.V.S.,
WASHINGTON, D. C.

THIS form of hernia occurs in the canine species more frequently than any other. It is characterized by the passage of a fold of peritoneum and visceral organs through the inguinal canal. The latter is very short, and its diameter varies according to the sex and conformation of the animal, being more pronounced in the female than in the male. The internal ring in the female is normally covered by parietal peritoneum, and consequently completely sealed.

The lesion is quite common in the female, and for some unknown reason occurs more frequently on the left side than on the right. In this connection it is worth remembering that normal descent of the testes in the male is said to be naturally later and more liable to deflection on the right side than on the left, suggesting that the mechanism of descent is more ample on the left. It is sometimes congenital in origin, but may not be detected until, the uterine cornua forming the contents and becoming gravid, it commences to enlarge with the growth of the contained fœtuses. It may often be observed to occur in animals after giving birth to litters sired by males very much their superiors in size. In these cases the ring seems to dilate in sympathy with the enormous distention of the abdominal cavity, its calibre being increased with the stretching of the wall. As soon as delivery is accomplished, part of the collapsed uterus falls through, carrying the peritoneum before it.

It is most common to find the round ligament together with the peritoneal fold by which it is connected with the broad ligament, and this is often the seat of deposit of a mass of fat. Some writers in describing inguinal hernia refer to this ligamentous tissue as omentum, evidently owing to the similarity between the two structures, but my investigations have shown me that it is a rare experience to discover omentum forming part of an inguinal hernia.

Next in frequency we find one or both uterine cornua, in part or whole, together with the broad and round ligaments, forming the contents of the sac. As already remarked, should pregnancy occur a rapidly growing enlargement soon becomes apparent. The increase in size may be first detected about one week after impregnation has taken place. Delivery of the fœtus *per vias naturales* is as a rule impossible, the inguinal ring forming an impassable constriction. Unless removed by operation of hysterotomy it dies and either macerates or decomposes and induces infective inflammation and gangrene of the maternal parts. Instances have been recorded, however, in which the young have been brought forth in a natural manner. Prangé (*Rec. de Méd. Vétér.*, 1844, p. 619) described a case of hernia of a portion of the uterus which contained three fœtuses, all of which were delivered naturally a few hours after birth of six others. Roell (cited by Fleming in *Vet. Obstetrics*) had a similar experience.

In one instance which I relieved by operation some time ago the entire uterus and one ovary together with the greater portion of the small intestine formed the protrusion. In this case the uterus was the seat of an enormous hydrometra formed from accumulated uterine secretions through occlusion of the cervical lumen by compression at the ring. Descent of other visceral organs may also occur. In one case recorded by Hobday (*Canine and Feline Surgery*) the sac was found to contain portions of the small intestine, pancreas, omentum, bladder, one uterine cornu, cæcum, and part of the rectum.

Adhesion of the pedicle of the protruding viscera to the borders of the ring is quite common, but it is rare that strangulation takes place when the uterus alone is concerned. Prudhomme (*Rec. de Méd. Vétér.*, 1844, p. 356) wrote of seeing such a case, which terminated fatally.

But when a knuckle of bowel slips into the sac the risk of strangulation is greatly increased.

The swelling is situated behind or beneath the inguinal mammæ. It may be of such dimensions as to reach the ground, when it is usually rendered sore by friction. Contrasted with neoplastic formations, it lies deeply, is more or less reducible, and decreases in size when the animal is placed in the dorsal position, when there is absence of adhesions. Its consistence varies according to the contents and the condition of the latter. A slight hernia is moderately firm and resistant. The presence of fluid, whether serous, mucous, or fœtal, may cause fluctuation. The forms as well as the movements of one or more fœtuses may sometimes be distinguished. By palpation a sort of pedicle can be recognized leading to the inguinal canal, and the circumference of the latter can often be plainly made out. If the bowel be involved, intestinal murmurs may be heard with the aid of a phonendoscope. The fact is worth remarking that nursing puppies as a rule refuse the teat or teats under which the hernia lies.

A word of caution as to exploration of enlargements in the inguinal region with instruments. Robb (*Journal of Comparative Pathology and Therapeutics*, vi., p. 281) recorded having observed an enlargement of the posterior mammary gland of the left side which fluctuated, showing the presence of liquid. An unsuccessful attempt was made to draw off the fluid with the aid of a canula. Finally the tumor was opened and hysterotomy performed and a fœtus removed. The mother died from peritonitis, which evidently had its origin at the site of puncture.

Raynard (*Traité complet de la Parturit. des Anim. Domest.*, i., p. 443) described a case in which the pregnant uterus protruded until it had lodged in the connective tissue immediately beneath the vulva. The owner supposing the enlargement to be an abscess opened it with a penknife, and thereby established a fistula. Raynard explored the tumor and found it to be the uterus containing a three- to four-months'-old fœtus. The hernia was irreducible owing to adhesions having formed between the uterus and connective tissue, and the animal died the following day.

In order to acquire a clear conception of the manner in which congenital hernia of the round and broad ligaments and the uterine cornua takes place, it will be necessary to review the prenatal development of the sexual organs.

In early fœtal life there exists an intra-abdominal glandular structure on each side of the lumbar region—the Wolffian body—the structure which gives rise to the reproductive gland of either sex. Connected with the Wolffian body are two tubular structures leading to the uro-genital sinus, the Wolffian and Muellerian ducts respectively, the former becoming the vas deferens and epididymis in the male, and the latter the oviduct, uterus, and vagina in the female. The Wolffian body is suspended from the abdominal roof by a duplicature of peritoneum from which a fold (the *plica gubernatrix*) is projected to the inguinal region. This fold subsequently becomes the gubernaculum testis in the male and the ligamentum teres in the female. It is invested throughout with peritoneum which forms a tubular sheath about it. The gubernaculum testis extends from the fœtal testicle through the inguinal canal to the scrotum, and beneath its sheath are found the cremasteric muscular fibres arising from the abdominal muscles.

The ligamentum teres has its origin at the anterior extremity of the horn and is prolonged through the inguinal canal to the subcutaneous fascia in the vicinity of the os pubis and sometimes the vulva. Like the gubernaculum, it also receives fibres from the abdominal muscles.

In the descent of the testis into the scrotum the gubernaculum with its enveloping cremasteric muscle and peritoneal sheath has the principal share in the formation of the vaginal process.

We have seen that in the female there is an arrangement of structures almost identical with that which serves to bring about migration of the testes in the male, and it would appear as if nature had almost intended the same phenomenon should occur in the female. As a matter of fact, we find evidence of an attempt on the part of nature to bring about migration of the female organs. When the muscular fibres of the ligamentum teres are sufficiently developed, the natural result is contraction, and there follows the formation of a pouch communicating directly with the peritoneal cavity, and constituting a sort of diverticulum as in males. This pouch forms the hernial sac in female inguinal hernia, and in proportion to its development and the potential contractility of the muscular fibres, predisposes the animal to this lesion. Goubaux (*Rec. de Méd. Vétér.*, 1858, p. 984) seems to have been the first veterinarian to have worked out the mechanism of inguinal hernia in the female.

